# A Reusable Impedimetric Aptasensor for Detection of Thrombin Employing a Graphite-Epoxy Composite Electrode

**DOI:** 10.3390/s120303037

**Published:** 2012-03-06

**Authors:** Cristina Ocaña, Mercè Pacios, Manel del Valle

**Affiliations:** Sensors and Biosensors Group, Department of Chemistry, Universitat Autònoma de Barcelona, Bellaterra 08193, Spain; E-Mails: cristina.ocana@uab.es (C.O.); merce.pacios@uab.es (M.P.)

**Keywords:** aptamer, thrombin, electrochemical impedance spectroscopy, labeless, adsorption

## Abstract

Here, we report the application of a label-free electrochemical aptasensor based on a graphite-epoxy composite electrode for the detection of thrombin; in this work, aptamers were immobilized onto the electrodes surface using wet physical adsorption. The detection principle is based on the changes of the interfacial properties of the electrode; these were probed in the presence of the reversible redox couple [Fe(CN)_6_]^3−^/[Fe(CN)_6_]^4−^ using impedance measurements. The electrode surface was partially blocked due to formation of aptamer-thrombin complex, resulting in an increase of the interfacial electron-transfer resistance detected by Electrochemical Impedance Spectroscopy (EIS). The aptasensor showed a linear response for thrombin in the range of 7.5 pM to 75 pM and a detection limit of 4.5 pM. The aptasensor was regenerated by breaking the complex formed between the aptamer and thrombin using 2.0 M NaCl solution at 42 °C, showing its operation for different cycles. The interference response caused by main proteins in serum has been characterized.

## 1. Introduction

Aptamers are artificial DNA or RNA oligonucleotides selected *in vitro* which have the ability to bind to proteins, small molecules or even whole cells, recognizing their target with affinities and specificities often matching or even exceeding those of antibodies [1]. Furthermore, the recognition process can be inverted and is stable in broad terms. Due to all these properties, aptamers can be used in a wide range of applications, such as therapeutics [2], molecular switches [3], drug development [4], affinity chromatography [5] and biosensors [6].

One of the most known and used aptamers is selective to thrombin, with the sequence 5′-GGTTGGTGTGGTTGG-3′. Thrombin is the last enzyme protease involved in the coagulation cascade, and converts fibrinogen to insoluble fibrin which forms the fibrin gel, both in physiological conditions and in a pathological thrombus [7]. Therefore, thrombin plays a central role in a number of cardiovascular diseases [8], and it is thought to regulate many processes such as inflammation and tissue repair at the blood vessel wall. Concentration levels of thrombin in blood are very low, and levels down to picomolar range are associated with disease; because of this, it is important to be able to assess this protein concentration at trace level, with high selectivity [9].

In previous years, there has been great interest in the development of aptasensors. Aptasensors are biosensors that use aptamers as the biorecognition element. Different transduction techniques such as optical [10], Atomic Force Microscope [11], electrochemical [12] and piezoelectric [13] variants have been reported. Recently, among the different electrochemical techniques available, the use of Electrochemical Impedance Spectroscopy (EIS) [14] has grown among studies [15,16]. EIS is rapidly developing as a reference technique for the investigation of bulk and interfacial electrical properties of any kind of solid or liquid material which is connected to or part of an appropriate electrochemical transducer. Impedance is a simple, high-sensitivity, low-cost and rapid transduction principle to follow biosensing events that take place at the surface of an electrode [17–19]. Moreover, apart from the detection of the recognition event when an immobilized molecule interacts with its target analyte, EIS can be used to monitor and validate the different sensing stages, including preparation of biosensor. Together with Surface Plasmon Resonance and the Quartz Crystal Microbalance, EIS is one of the typical transduction techniques that do not require labelled species for detection.

In the present communication, we report the application a label-free electrochemical aptasensor for the detection of thrombin using graphite-epoxy composite electrodes (GEC). This platform is of general use in our laboratories and has been already extensively studied and applied for amperometric, enzymatic, immuno- and genosensing assays [20,21]. The uneven surface of the graphite-epoxy electrode allows the immobilization of the aptamer onto its surface by simple wet physical adsorption. Afterwards, the electrode surface may be renewed after each experiment by polishing with abrasive paper. The transduction principle used is based on the change of electron-transfer resistance in the presence of the [Fe(CN)_6_]^3−^/[Fe(CN)_6_]^4−^ redox couple, which can be measured by EIS. The proposed aptasensor showed appropriate response behaviour values to determine thrombin in the picomolar range. Moreover, this proposed method has some advantages such as high sensitivity, simple instrumentation, low production cost, fast response, portability and what’s more, the biosensor has been shown to be easily regenerated by wet procedures.

## 2. Experimental

### 2.1. Chemicals

Potassium ferricyanide K_3_[Fe(CN)_6_], potassium ferrocyanide K_4_[Fe(CN)_6_], potassium dihydrogen phosphate, sodium monophosphate and the target protein thrombin (Thr), were purchased from Sigma (St. Louis, MO, USA). Poly(ethylene glycol) (PEG), sodium chloride and potassium chloride were purchased from Fluka (Buchs, Switzerland). All reagents were analytical reagent grade. All-solid-state electrodes (GECs) were prepared using 50 μm particle size graphite powder (Merck, Darmstadt, Germany) and Epotek H77 resin and its corresponding hardener (both from Epoxy Technology, Billerica, MA, USA). The aptamer (AptThr) used in this study, with sequence 5′-GGTTGGTGTGGTTGG-3′, was prepared by TIB-MOLBIOL (Berlin, Germany). All solutions were made up using MilliQ water from MilliQ System (Millipore, Billerica, MA, USA). The buffer employed was PBS (187 mM NaCl, 2.7 mM KCl, 8.1 mM Na_2_HPO_4_·2H_2_O, 1.76 mM KH_2_PO_4_, pH 7.0). Stock solutions of aptamer and thrombin were diluted with sterilized and deionised water, separated in fractions and stored at −20 °C until used.

### 2.2. Apparatus

AC impedance measurements were performed with an IM6e Impedance Measurement Unit (BAS-Zahner, Kronach, Germany) and Autolab PGStat 20 (Metrohm Autolab B.V, Utrecht, The Netherlands). Thales (BAS-Zahner) and Fra (Metrohm Autolab) software, respectively, were used for data acquisition and control of the experiments. A three electrode configuration was used to perform the impedance measurements: a platinum-ring auxiliary electrode (Crison 52–67 1, Barcelona, Spain), an Ag/AgCl reference electrode and the constructed GEC as the working electrode. Temperature-controlled incubations were done using an Eppendorf Thermomixer 5436.

### 2.3. Preparation of Working Electrodes

**Figure 1.**
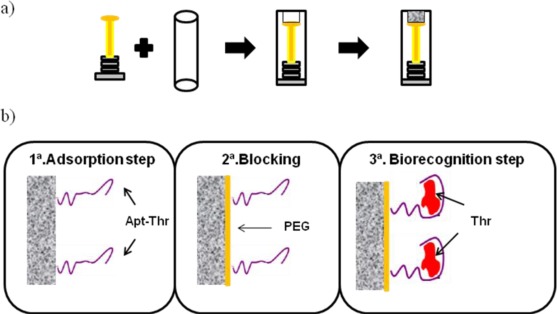
(**a**) Scheme of the manufacture of graphite-epoxy composite electrodes, (**b**) Steps of the biosensing procedure.

Graphite epoxy composite (GEC) electrodes used were prepared using a PVC tube body (6 mm i.d.) and a small copper disk soldered at the end of an electrical connector, as shown on Figure 1(a). The working surface is an epoxy-graphite conductive composite, formed by a mixture of graphite (20%) and epoxy resin (80%), deposited on the cavity of the plastic body [15,16]. The composite material was cured at 80 °C for 3 days. Before each use, the electrode surface was moistened with MilliQ water and then thoroughly smoothed with abrasive sandpaper and finally with alumina paper (polishing strips 301044-001, Orion) in order to obtain a reproducible electrochemical surface.

### 2.4. Procedure

The analytical procedure for biosensing consists of the immobilization of the aptamer onto the transducer surface using a wet physical adsorption procedure, followed by the recognition of the thrombin protein by the aptamer via incubation at room temperature. The scheme of the experimental procedure is represented in Figure 1(b), with the steps described in more detail below.

#### 2.4.1. Aptamer Adsorption

First, 160 μL of aptamer solution in MilliQ water at the desired concentration was heated at 80–90 °C for 3 min to promote the loose conformation of the aptamer. Then, the solution was dipped in a bath of cold water and the electrode was immersed in it, where the adsorption took place at room temperature for 15 min with soft stirring. Finally, this was followed by two washing steps using PBS buffer solution for 10 min at room temperature, in order to remove unadsorbed aptamer.

#### 2.4.2. Blocking

After aptamer immobilisation, the electrode was dipped in 160 μL of PEG 40 mM for 15 min at room temperature with soft stirring to minimize any possible nonspecific adsorption. This was followed by two washing steps using PBS buffer solution for 10 min.

#### 2.4.3. Label-Free Detection of Thrombin

The last step is the recognition of thrombin by the immobilized aptamer. For this, the electrode was dipped in a solution with the desired concentration of thrombin. The incubation took place for 15 min at room temperature with soft stirring. After that, the biosensor was washed twice with PBS buffer solution for 10 min at room temperature to remove nonspecific adsorption of protein.

#### 2.4.4. Regeneration of Aptasensor

Finally, to regenerate the aptasensor, the aptamer-thrombin complex must be broken. For this, the electrode was dipped in a 2 M NaCl, heated at 42 °C while stirring for 20 min. Afterwards, the electrode was washed twice with PBS buffer solution for 10 min.

### 2.5. Impedimetric Measurements

**Figure 2.**
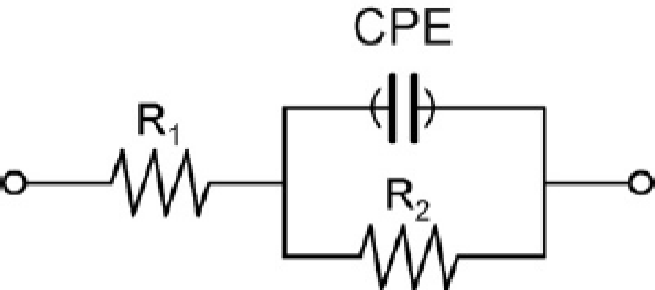
Equivalent circuit used for the data fitting. R_1_ is the resistance of the solution, R_2_ is the electron-transfer resistance and CPE, the capacitive contribution, in this case as a constant phase element.

Impedimetric measurements were performed in 0.01 mM [Fe(CN)_6_]^3−/4−^ solution prepared in PBS at pH 7. The electrodes were dipped in this solution and a potential of +0.17 V (*vs.* Ag/AgCl) was applied. Frequency was scanned from 10 kHz to 50 mHz with a fixed AC amplitude of 10 mV. The impedance spectra were plotted in the form of complex plane diagrams (Nyquist plots, −Z_im_ *vs.* Z_re_) and fitted to a theoretical curve corresponding to the equivalent circuit with Z_view_ software (Scribner Associates Inc., USA). In the equivalent circuit shown in Figure 2, the parameter R_1_ corresponds to the resistance of the solution, R_2_ is the charge transfer resistance (also called R_ct_) between the solution and the electrode surface, whilst CPE is associated with the double-layer capacitance (due to the interface between the electrode surface and the solution). The use of a constant phase element (CPE) instead of a capacitor is required to optimize the fit to the experimental data, and this is due to the nonideal nature of the electrode surface [14]. The parameters of interest in our case are the electron-transfer resistance (R_ct_) and the chi-square (χ^2^). The first one was used to monitor the electrode surface changes, while χ^2^ was used to measure the goodness of fit of the model. In all cases the calculated values for each circuit were <0.2, much lower than the tabulated value for 50 degrees of freedom (67.505 at 95% confidence level). In order to compare the results obtained from the different electrodes used, and to obtain independent and reproducible results, relative signals are needed [16]. Thus, the Δ_ratio_ value was defined according to the following equations:

$$\Delta_{\text{ratio}}=\Delta_{s}/\Delta_{p}$$

$$\Delta_{s}=R_{\text{ct}\;(\text{AptThr-Thr})}-R_{\text{ct}\;(\text{electrode-buffer})}$$

$$\Delta_{p}=R_{\text{ct}\;(\text{AptThr})}-R_{\text{ct}\;(\text{electrode-buffer})}$$

where R_ct(AptThr-Thr)_ was the electron transfer resistance value measured after incubation with the thrombin protein; R_ct(AptThr)_ was the electron transfer resistance value measured after aptamer inmobilitation on the electrode, and R_ct(electrode-buffer)_ was the electron transfer resistance of the blank electrode and buffer.

## 3. Results and Discussions

### 3.1. Optimization of Electrode Surface

First of all, the concentration of aptamer and PEG immobilized onto the electrode surface were optimized separately by building its response curves. For this, increasing concentrations of AptThr and PEG were used to carry out the immobilization, evaluating the changes in the Δ_p_.

**Figure 3.**
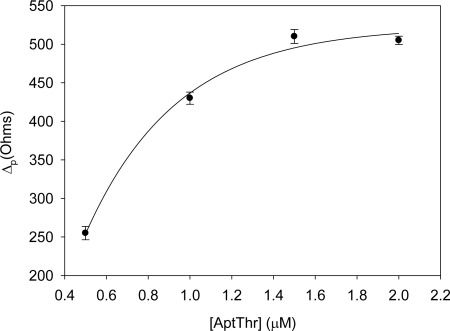
Optimization of the concentration of Apt-Thr. Uncertainty values corresponding to replicated experiments (n = 5).

Figure 3 shows the curve of AptThr adsorption onto the electrode surface. It can be observed that the difference in resistance (Δ_p_) increased up to a value. This is due to the physical adsorption of the aptamer onto the electrode surface, which followed a Langmuir isotherm; in it, the variation of R_ct_ increases to reach a saturation value, chosen as the optimal concentration. This value corresponded to a concentration of aptamer of 1 μM.

**Figure 4.**
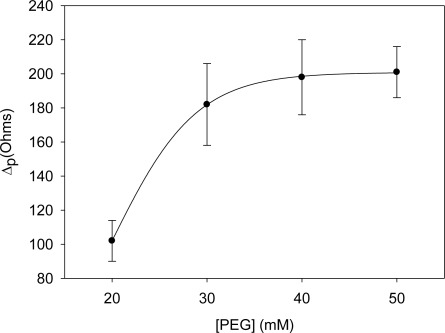
Optimization of the concentration of the blocking agent, PEG. Uncertainty values corresponding to replicated experiments (n = 5).

To minimize any possible nonspecific adsorption onto the electrode surface, polyethylene glycol (PEG) was used as the blocking agent. As shown in Figure 4, and like the previous case, there was an increase in resistance until the value of 40 mM of PEG where the saturation value is reached. Therefore, the optimal concentration of blocking agent was chosen as 40 mM.

### 3.2. Detection of Thrombin

**Figure 5.**
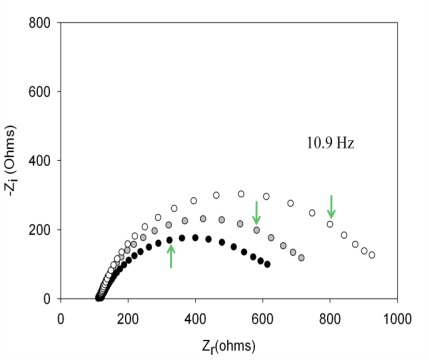
Nyquist Diagram of: (**a**) Electrode-buffer •, (**b**) Aptamer of thrombin (AptThr) 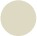, and (**c**) AptThr-Thr ○ 10 pM [Thr]. The arrow in each spectrum denotes the frequency (AC) of 10.9 Hz.

With the optimized concentrations of aptamer and PEG and following the above experimental protocol for the detection of thrombin, the aptasensor response was initially evaluated. The aptamer of thrombin forms a single-strand oligonucleotide, a chain that recognizes the protein by a three-dimensional folding (quadriplex). During this folding, weak interactions between the aptamer and protein of the host-guest type are created, leading to complex AptThr-Thr [22]. One example of the obtained response after each biosensing step is shown in Figure 5. As can be seen, resistance R_ct_ between the electrode surface and the solution is increased. This fact is due to the effect on the kinetics of the electron transfer redox marker [Fe(CN)_6_]^3−^/[Fe(CN)_6_]^4−^ which is delayed at the interface of the electrode, mainly caused by steric hindrance and electrostatic repulsion presented by the complex formed.

**Figure 6.**
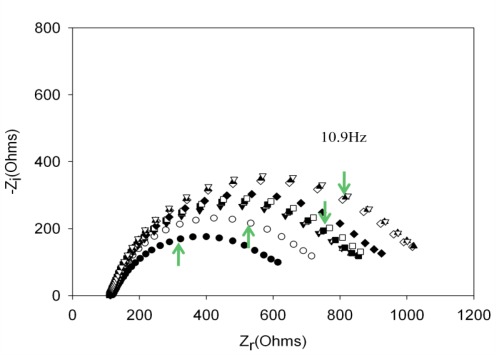
Nyquist diagrams for different concentrations of thrombin. • Electrode-buffer, ○ AptThr, ▾ 1 × 10^−12^ M [Thr], ▵ 2.5 × 10^−12^ M [Thr], ▪ 5.5 × 10^−12^ M [Thr], □ 7.5 × 10^−12^ M [Thr], ♦ 1 × 10^−11^ M [Thr], ⋄ 5 × 10^−11^ M [Thr], ▴ 7.5 × 10^−11^ M [Thr], ▿ 1 × 10^−10^ M [Thr]. The arrow in each spectrum denotes the frequency (AC) of 10.9 Hz.

**Figure 7.**
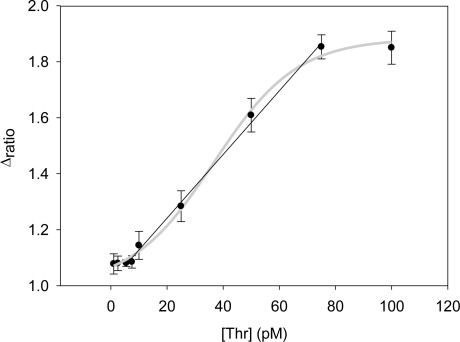
Calibration curve *vs.* thrombin concentration. (Δ_ratio_ = Δ_s_ /Δ_p_; Δ_s_ = R_ct(AptThr-Thr)_ − R_ct (electrode-buffer)_; Δ_p_ = R_ct (AptThr)_ − R_ct(electrode-buffer)_).

Performing new experiments with solutions containing different amount of thrombin, the calibration curve was built. Figure 6 shows the evolution of the Nyquist diagrams for the calibration of the aptasensor. There is a correct recognition of the protein by the aptamer; as by increasing thrombin concentration, the interfacial electron transfer resistance between the electrode surface and solution also increases, until reaching saturation. To evaluate the linear range and detection limit of the AptThr-Thr system, the calibration curve was built, representing the analytical signal expressed as Δ_ratio_ *vs.* the protein concentration (Figure 7). As can be seen, a sigmoidal trend is obtained, where the central area could be approximated to a straight line, with a linear range from 7.5 pM to 75 pM for the protein. Moreover, a good linear relationship (r^2^ = 0.9981) between the analytical signal (Δ_ratio_) and the thrombin concentration in this range was obtained, according to the equation: Δ_ratio_ = 1.013 + 1.106 × 10^10^ [Thr]. The EC_50_ was estimated as 44 pM and the detection limit, calculated as three times the standard deviation of the intercept obtained from the linear regression, was 4.5 pM. The reproducibility of the method showed a relative standard deviation (RSD) of 7.2%, obtained from a series of 5 experiments carried out in a concentration of 75 pM Thr. These are satisfactory results for the detection of thrombin in real samples, given this level is exactly the concentration threshold when forming thrombus [9].

### 3.3. Selectivity of Aptasensor

Thrombin is present in blood serum, a complex sample matrix, with hormones, lipids, blood cells and other proteins [23]. To study the selectivity of the system, we evaluated the response of proteins typically present in serum such as fibrinogen, immunoglobulin G and albumin. In the first case, we tested albumin protein, which is found in serum at a level from 3,500 to 5,000 mg/dL [24], representing more than 60% of the total protein present. To perform the test, the highest concentration in serum was used, that is 5,000 mg /dL. When the aptamer was incubated with this protein, electron interfacial resistance did not increase, in this case it was observed a slight decrease. Afterwards, when the aptamer was incubated with thrombin, an increase in the resistance, and of expected magnitude, was observed. Therefore, it was proved that albumin was not recognized by the AptThr, and it did not interfere with aptamer-thrombin system.

In the second case, fibrinogen was evaluated as an interfering protein. Fibrinogen is a fibrillar protein involved in the blood clotting process. By the action of thrombin, fibrin is degraded and results in the formation of a clot [25]. This protein is present in human serum in a concentration range of 200 to 400 mg/dL [26]. It was observed that the electron interfacial resistance increased as a result of some type of recognition by the AptThr. Therefore, this protein could act as an interference for the system.

In the last case, generic immunoglobulin G (IgG) was used. IgG is a globular protein that is synthesized in response to the invasion of any bacteria, virus or fungi. It is present in human serum over a range of concentrations from 950 mg/dL to 1,550 mg/dL in serum, with a normal value of 1,250 mg/dL. IgG also acted as interferent, which is proved from the increase of the resistance R_ct_. This increase, as it also happened in the case of fibrinogen, may be due to some biological interaction between the aptamer and these proteins, not yet described. In the last two cases, the addition of thrombin to the system increased the interfacial resistance between the electrode and the surface; this fact could be due to some phenomenon of partial displacement between thrombin and protein interferents that could take place.

**Figure 8.**
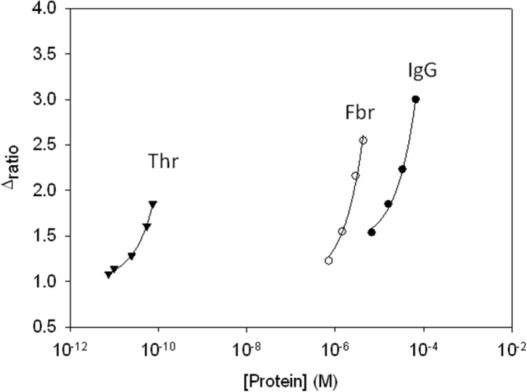
Response to proteins evaluated: IgG (•), Fbr (○) and Thr (▾).

To evaluate the sensitivity of the aptasensor we compared the calibration plots for the different proteins. Table 1 summarizes the parameters of the calibration curve of each protein and thrombin, as well as their respectives slopes and detection limits. The aptasensor showed the highest sensitivity for its target molecule, thrombin, with its slope being 6 orders of magnitude greater than the slope for IgG and five orders of magnitude more than the one for fibrinogen, as shown in Figure 8. Therefore, it was demostrated that the aptasensor showed a much higher sensitivity to Thr, regarding potential interfering proteins, which displayed this effect due to the high level of concentration in which they are present.

### 3.4. Regeneration of Aptasensor

**Figure 9.**
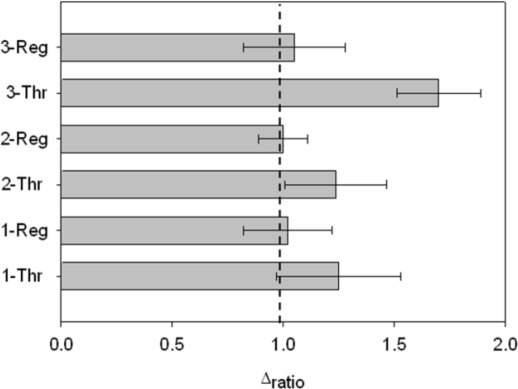
Signals obtained for three consecutive processes of regeneration. [Thr] used: 7.5 pM (1 and 2), and 75 pM (3). Uncertainty values corresponding to replicated experiments (n = 3).

Finally, it was possible to regenerate the aptasensor by dissociating the AptThr-Thr complex, formed by weak interactions. It was achieved by stirring the aptasensor in saline media and increasing the temperature (42 °C). In this way, to show regeneration, three sensing cycles were performed with a blank measure in between each. Thrombin was added to the media and an increase of R_ct_ due to complex formation AptThr-Thr was recorded. Then, by adding a saline buffer, increasing the temperature and stirring, the complex dissociated and resistance decreased to the baseline value of the correspondent (AptThr), and so on. Values were calculated as Δ_ratio_ on every step of the process and represented in the bar chart as shown in Figure 9. In the third incubation with Thr, Δ_ratio_ was increased more than in the other incubations, this was because it was incubated with a higher concentration. This type of regeneration may be an alternative to the polishing surface renewal, a typical feature of graphite-epoxy composite electrodes; an important advantage is that it regenerates the electrode surface without removing the immobilized aptamer on the electrode surface, which means that their use is largely facilitated and that cost of each analysis is reduced drastically.

## 4. Conclusions

In conclusion, this work reports a simple, label-free and reusable aptasensor for detection of thrombin. The immobilized aptamer retained its bioactivity and could be used for recognition of the target substrate. The aptamer immobilization step or the recognition event modified the electron transfer kinetics of the redox probe at the electrode interface, which was examined by EIS.

The described aptasensor, based on physical adsorption of the aptamer, showed a low detection limit, good range of concentration for thrombin detection and high sensitivity. The interference produced by serum proteins, fibrinogen and immunoglobulin G, not described before, showed some limitations in the operation of the aptasensor, although usable given the concentration excess at which they manifest. In addition, the aptasensor can be regenerated by dissociating the complex formed between the aptamer and thrombin protein .This fact presents an alternative to polishing regeneration and reduces the cost of analysis.

## Figures and Tables

**Table 1.** Summary of calibration results for thrombin and other major proteins presents in serum.

**Protein**	**Regression Line**	**Sensitivity (M^−1^)**	**Detection limit**	**Typical conc. in serum**
Thr	Δ_ratio_ = 1.013 + 1.106 × 10^10^ [Thr]	1.106 × 10^10^	4.5 pM	0
Fbr	Δ_ratio_ = 1.007 + 3.698 × 10^5^ [Fbr]	3.698 × 10^5^	2 μM	6–12 μM
IgG	Δ_ratio_ =1.424 + 2.385 × 10^4^ [IgG]	2.385 × 10^4^	10 μM	60–100 μM
Albumin	No response	−	−	0.52–0.75 mM
